# Undergraduate general medicine education in Japan: A nationwide cross‐sectional survey of medical trainees' perspectives

**DOI:** 10.1002/jgf2.752

**Published:** 2024-11-10

**Authors:** Hirohisa Fujikawa, Hidetaka Tamune, Yuji Nishizaki, Kiyoshi Shikino, Taro Shimizu, Yu Yamamoto, Yasuharu Tokuda

**Affiliations:** ^1^ Center for General Medicine Education School of Medicine, Keio University Tokyo Japan; ^2^ Department of Medical Education Studies, International Research Center for Medical Education, Graduate School of Medicine The University of Tokyo Tokyo Japan; ^3^ Department of Psychiatry and Behavioral Science Juntendo University Graduate School of Medicine Tokyo Japan; ^4^ Division of Medical Education Juntendo University School of Medicine Tokyo Japan; ^5^ Department of Community‐Oriented Medical Education, Graduate School of Medicine Chiba University Chiba Japan; ^6^ Department of Diagnostic and Generalist Medicine Dokkyo Medical University Hospital Tochigi Japan; ^7^ Division of General Medicine, Center for Community Medicine Jichi Medical University Tochigi Japan; ^8^ Muribushi Okinawa for Teaching Hospitals Okinawa Japan; ^9^ Tokyo Foundation for Policy Research Tokyo Japan

**Keywords:** general medicine, generalism, model core curriculum, undergraduate medical education

## Abstract

**Background:**

The 2022 revised version of the Model Core Curriculum (MCC) for Medical Education in Japan includes “generalism” as a new expertise quality and ability, based on the results of surveys of experts in health professions education. However, the perspectives of medical trainees under the pre‐2022 MCC revision were under‐examined. Here, we investigated what these trainees felt they had learned about general medicine (GM)‐related topics.

**Methods:**

We performed a nationwide cross‐sectional study using an anonymous online questionnaire, which was developed with reference to the 2022 revised MCC. The questionnaire consisted of 14 items. For all items, we asked, “Did you learn enough during medical school?” Respondents were asked to respond on a 5‐point Likert scale (from 1 = strongly disagree to 5 = strongly agree).

**Results:**

Three hundred and eighty‐six participants (response rate 55.4%) were included in the analysis. For the item “Behavioral science,” the number of participants who chose “3 = neither agree nor disagree” was highest, at 171 (44.3%) and with an average of 3.28, indicating that this item was perceived as insufficiently studied. Approximately half of the participants chose “4 = agree” for all items other than “Behavioral science.”

**Conclusions:**

The study suggested that behavioral science may be underlearned among medical trainees of the pre‐2022 MCC generation. Medical educators in Japan should formulate curricula in accordance with the 2022 revision MCC and improve curricula regarding behavioral science. Future research should survey the generation of trainees who receive 2022 revision MCC‐compliant medical education; comparison of results with those of this study would be valuable in examining the effects of the revised guideline and inform international medical educators.

## 1 INTRODUCTION

The healthcare environment has recently faced major challenges. Prominent examples are the rapid aging of the population and the increase in the number of patients with multimorbidity, which are major concerns worldwide.^1^ Further, although increased specialization of physicians is a global phenomenon,^2^ overspecialization can result in potential concerns; in patients with undiagnosed symptoms or multiple problems, seeing a specialist in a single area may not provide appropriate care.^3^ Accordingly, the importance of general medicine (GM) is becoming increasingly evident. In fact, a substantial body of literature shows that primary care, including GM, is crucial to improving population health,^4^ healthcare quality and equity,^5^, ^6^ and reducing healthcare costs.^7^ The World Health Organization has also described the role of GM as indispensable.^8^ For clarity, although a recent literature search study revealed that the definition of GM is unclear in Japan,^9^ in this paper, we defined GM physicians as physicians who provide comprehensive care, taking a multisystemic and holistic approach to patients, considering their lifestyle and broader social and/or family context, rather than being limited to the silo of a particular medical specialty.^10^ In addition, although medical specialties that provide comprehensive care include various specialties such as GM, family medicine, general internal medicine, and primary care,^11^ and these terms are often used interchangeably in Japan, we use the term GM in this article.

Undergraduate GM education is also critical for three reasons. First, as noted above, GM involvement is associated with better outcomes (e.g., better population health, better healthcare quality, and lower costs), and the concept should be taught prior to graduation, because many physicians cannot learn about the concept after graduation because of time limitations. Effective GM education is expected to lead to better patient outcomes in the future.^10^ Second, teaching GM to medical students has a positive impact on future career choices.^12^ This is also important given the global shortage of GM physicians.^13^ Third, GM appears to be an ideal setting for medical undergraduates to learn the basic competencies and skills as physicians required to deal with conditions they commonly treat. Learning from GM physicians would include history and physical examination, as well as items such as consultation skills, communication skills, disease prevention, health promotion, and person‐centered care.^14^, ^15^

The level of involvement and contribution of GM in the education of medical students varies by country. Previous reviews regarding teaching and learning in ambulatory care in North America indicated the GM's positive contribution to undergraduate medical education.^16^, ^17^, ^18^ Conversely, medical educators in Europe appear to struggle with undergraduate GM education. The provision of undergraduate GM education remains low in many European countries.^19^ In some European countries, GM constitutes slightly greater than 10% of the clinical education content.^20^ Undergraduate GM education received less coverage or is neglected entirely in other European countries.^21^ To address this challenge, the European Academy of Teachers in General Practice/Family Medicine has developed pan‐European guidelines for undergraduate GM education that would strengthen GM education at the local, national, and international levels.^21^

In Japan, a 2022 update of the Model Core Curriculum (MCC) for Medical Education has been adopted as a guideline for undergraduate medical education by all 82 medical schools in Japan. Approximately two‐thirds of each medical school's curriculum should be aligned with the MCC.^22^ The 2022 revised guideline incorporates “generalism” as a new expertise quality and ability.^10^, ^23^ This version of the MCC was released in November 2022 and introduced for students who enroll in the 2024 academic year. By introducing the new “generalism”‐related competencies within the MCC, all medical students will obtain the opportunity to develop and learn “generalism,” thus helping to meet the anticipated future needs of society and improving patients' well‐being.^10^ Additionally, improving undergraduate GM education may change the current situation in medical practice, where there is a deeply entrenched hierarchical structure in which organ‐specific specialists may show disrespect to GM physicians^24^; future organ‐specific specialists could foster mutual trust and respect between generalists and specialists through GM education, promoting intraprofessional learning and collaborative practice.^25^, ^26^, ^27^

The decision to incorporate “generalism” in the 2022 revision of the MCC is based on the results of a questionnaire‐based survey conducted by the Japan Society for Medical Education in those in charge of medical education at universities across Japan,^28^ as well as subsequent interviews conducted by the MCC project team with experts on healthcare in society (unpublished). These surveys showed that the last updated version (i.e., 2016 revision) of the MCC lacked certain educational content regarding generalism, including a comprehensive attitude in approach towards patients, difference between disease and illness, and salutogenesis.^28^ Accordingly, the MCC was revised to reflect the perspectives of the medical educators, and it is expected that improved GM education will likely be provided in Japan.

Meanwhile, there appears to be a lack of research on an important stakeholder: to our knowledge, medical students of the pre‐2022 MCC revision generation have not been surveyed to determine the extent to which they felt they learned GM‐related content. Therefore, the aim of this study was to examine the extent to which they felt they had learned content related to GM. We also aimed to explore individual and university factors associated with the degree of GM learning. The findings of the study would be insightful because it examines the perspectives of a neglected stakeholder, the medical student, on the state of GM education in Japan at the time prior to the guideline revision. Moreover, future surveys of the generation of students taught the 2022 revision MCC would allow the comparison of results with those of the present study, thereby validating or otherwise the effectiveness of the revised guideline and in turn providing deep knowledge for medical educators in Japan and abroad.

## 2 METHODS

### 2.1 Study design, setting, and participants

We performed the study under a cross‐sectional design throughout Japan from April 5 to 30, 2024. An anonymous online questionnaire was distributed to postgraduate Year 1 resident physicians who took the General Medicine In‐Training Examination postgraduate “Year‐0” (GM‐ITE PGY‐0). The generation that entered early residency training in April 2024 is the generation that received undergraduate medical education under the previously revised (i.e., 2016 revision) MCC. In the Japanese medical education system, the residency program starts in April, coincident with the new fiscal year of medical institutions.^22^ Accordingly, the timing of the survey was just after graduation from medical school, which was considered an appropriate time to ask about the content of education during medical school. The GM‐ITE PGY‐0 was first implemented in 2018 by a nonprofit organization, the Japan Organization of Advancing Medical Education Program (JAMEP). The GM‐ITE PGY‐0 examination is held throughout Japan and is administered shortly after the start of clinical training, thus captures data on trainees at the beginning of their residency.

All eligible study participants were asked to participate in our study immediately after the GM‐ITE PGY‐0. They read the research description document, which informed them about the anonymous and voluntary nature of the study. We included only participants who showed willingness to participate.

### 2.2 Measurements

The authors developed a questionnaire to evaluate how well undergraduate GM medicine education was provided (supplementary file—Data S1). First, the first author repeatedly reviewed the “generalism” section of the 2022 revised MCC.^10^, ^23^ This section consists of four components of quality/ability (“holistic perspectives and approaches,” “community perspectives and approaches,” “life perspectives and approaches,” and “social perspectives and approaches”), and each component has several competencies.^10^, ^23^ Second, the first author then drafted the questionnaire with reference to this structure. Third, all authors iteratively discussed and revised the draft, which led to consensus. The final version of the questionnaire consisted of 14 items (Table 1). For all items, we asked, “Did you learn enough during medical school?” All items were rated on a 5‐point Likert scale, ranging from 1 = strongly disagree to 5 = strongly agree.

**TABLE 1:** The details of the questionnaire.

Question	Category measures	Item
Did you learn enough about the following items during medical school?	Holistic perspectives and approaches	Transdisciplinary care Comprehensive perspectives on biological, psychological, and social issues Patient‐centered medicine Evidence‐based medicine Behavioral science Palliative care
	Community perspectives and approaches	Basic concepts in primary care Primary care in the community Provision of primary care according to medical resources Primary care at home
	Life perspectives and approaches	Care with an awareness of life processes (life cycle and life events)
	Social perspectives and approaches	Health in medical, cultural, and social contexts Social sciences (cultural anthropology and sociology (primarily medical anthropology and medical sociology))
In general, did you learn enough about general medicine during medical school?	Overall	

### 2.3 Statistical analysis

First, descriptive statistics were conducted. The distribution of responses for the 14 items as well as the mean (standard deviation) are reported.

Second, we performed multivariable linear regression analysis to explore factors associated with the degree of GM learning. To assess GM‐related topics comprehensively and for each of the four components of quality/ability listed in the MCC, we used the overall mean of the 13 items and the mean of each of the four components as outcome variables. We included the following individual or university factors that might be associated with the degree of GM learning as explanatory variables: participant gender (male, female, or others), participant age (24, 25, 26, or above 26 years old), university type (private university or national public university), and university location (rural or urban). Prior to the analysis, we checked the intraclass correlation coefficient (ICC) to examine whether multilevel analysis was needed or not.^29^ Since undergraduate GM education is provided by each university, it was necessary to examine whether there was clustering by the universities from which residents came. In our dataset, the ICC was less than 0.1 for the outcome variables; accordingly, because the clustering effect was considered small,^29^ we concluded that multilevel analysis was not needed.

We excluded participants from foreign medical schools because this study aimed at assessing Japanese undergraduate medical education. We also excluded participants with missing data (i.e., complete case analysis). A two‐tailed p‐value less than 0.05 was considered statistically significant. We used SPSS version 29.0 (IBM Corp.) for all statistical analysis.

### 2.4 Ethical considerations

Before participating in the study, all participants in this study read the document that fully explained about the study. It included a description about the anonymity and voluntary nature of this study. The participants' questionnaire responses were analyzed anonymously. The study was conducted according to the ethical standards and principles of the Declaration of Helsinki. It was approved by the ethics committee of JAMEP (approval number: 23–35).

## 3 RESULTS

**FIGURE 1:**
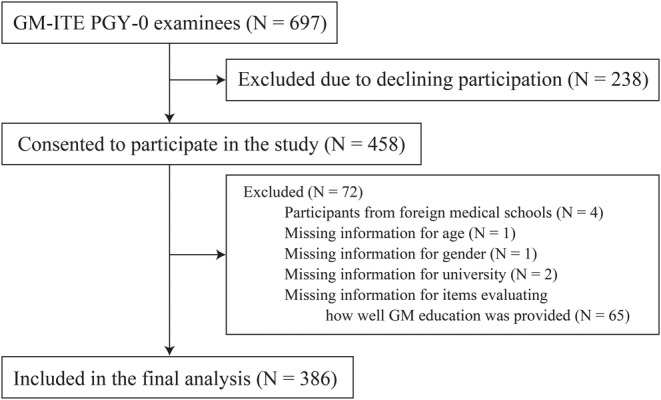
Participant flowchart. GM‐ITE PGY‐0, General Medicine In‐Training Examination postgraduate Year‐0.

Of the 697 resident physicians who took the GM‐ITE PGY‐0, 458 consented to participate. We excluded 72 participants with missing data and/or from foreign medical schools. The data of the remaining 386 participants (55.4%) were included in the analysis (Figure 1). Table 2 summarizes the participant profile. They were from 74 medical schools across Japan. The majority of participants were male (256, 66.3%), were from national public universities (258, 66.8%), and were from rural universities (234, 60.6%). 156 (40.4%) were 24 years old.

**TABLE 2:** Participant profile of the study (*N* = 386).

Characteristics	Value
Gender, *n* (%)	
Male	256 (66.3)
Female	126 (32.6)
Others	4 (1.0)
Age, *n* (%)	
24	156 (40.4)
25	112 (29.0)
26	63 (16.3)
≥ 27	55 (14.2)
University type, *n* (%)	
National or public	258 (66.8)
Private	128 (33.2)
University location, *n* (%)	
Rural	234 (60.6)
Urban	152 (39.4)

Responses to the questionnaire are shown in Table 3. Approximately half of the participants chose “4 = agree” for all items other than “Behavioral science.” The highest number of respondents chose “3 = neither agree nor disagree” for the item “Behavioral science,” at 171 (44.3%), with an average of 3.28, indicating that this item was perceived as insufficiently learned by the respondents compared to the other items.

**TABLE 3:** Responses to the questionnaire (*N* = 386).

Items	Responses, *n* (%)					Mean (standard deviation)
	1 = strongly disagree	2 = disagree	3 = neither agree nor disagree	4 = agree	5 = strongly agree	
Transdisciplinary care	4 (1.0)	44 (11.4)	146 (37.8)	166 (43.0)	26 (6.7)	3.43 (0.82)
Comprehensive perspectives on biological, psychological, and social issues	1 (0.3)	39 (10.1)	124 (32.1)	198 (51.3)	24 (6.2)	3.53 (0.77)
Patient‐centered medicine	1 (0.3)	14 (3.6)	85 (22.0)	219 (56.7)	67 (17.4)	3.87 (0.74)
Evidence‐based medicine	4 (1.0)	18 (4.7)	107 (27.7)	215 (55.7)	42 (10.9)	3.71 (0.76)
Behavioral science	7 (1.8)	50 (13.0)	171 (44.3)	143 (37.0)	15 (3.9)	3.28 (0.81)
Palliative care	5 (1.3)	55 (14.2)	133 (34.5)	169 (43.8)	24 (6.2)	3.39 (0.85)
Basic concepts in primary care	3 (0.8)	23 (6.0)	99 (25.6)	228 (59.1)	33 (8.5)	3.69 (0.74)
Primary care in the community	7 (1.8)	28 (7.3)	100 (25.9)	208 (53.9)	43 (11.1)	3.65 (0.84)
Provision of primary care according to medical resources	9 (2.3)	35 (9.1)	147 (38.1)	170 (44.0)	25 (6.5)	3.43 (0.84)
Primary care at home	14 (3.6)	47 (12.2)	117 (30.3)	187 (48.4)	21 (5.4)	3.40 (0.90)
Care with an awareness of life processes (life cycle and life events)	6 (1.6)	33 (8.5)	127 (32.9)	196 (50.8)	24 (6.2)	3.52 (0.80)
Health in medical, cultural, and social contexts	4 (1.0)	29 (7.5)	135 (35.0)	196 (50.8)	22 (5.7)	3.53 (0.76)
Social sciences (cultural anthropology and sociology (primarily medical anthropology and medical sociology))	10 (2.6)	53 (13.7)	144 (37.3)	162 (42.0)	17 (4.4)	3.32 (0.86)
Overall	9 (2.3)	55 (14.2)	127 (32.9)	175 (45.3)	20 (5.2)	3.37 (0.87)

We explored whether any individual or hospital factors were associated with the degree of GM learning via the multivariable linear regression analysis. The results are shown in Table 4. No significantly associated factors were identified for any outcome variable.

**TABLE 4:** The result of the multivariable linear regression analysis to explore the factors that were associated with the degree of GM learning.

	Adjusted mean difference	95% CI
Total (Mean 3.52, SD 0.58)^a^		
Gender		
Male	Ref.	Ref.
Female	0.01	−0.12 to 0.13
Others	0.05	−0.54 to 0.63
Age		
24	Ref.	Ref.
25	0.09	−0.06 to 0.23
26	0.01	−0.17 to 0.18
≥ 27	−0.09	−0.27 to 0.10
University type		
National or public	Ref.	Ref.
Private	0.06	−0.07 to 0.19
University location		
Rural	Ref.	Ref.
Urban	0.03	−0.10 to 0.15
Holistic perspectives and approaches (Mean 3.54, SD 0.59)^a^		
Gender		
Male	Ref.	Ref.
Female	0.01	−0.11 to 0.14
Others	0.03	−0.56 to 0.62
Age		
24	Ref.	Ref.
25	0.04	−0.11 to 0.19
26	−0.01	−0.19 to 0.16
≥ 27	−0.12	−0.31 to 0.07
University type		
National or public	Ref.	Ref.
Private	0.08	−0.05 to 0.21
University location		
Rural	Ref.	Ref.
Urban	0.07	−0.06 to 0.19
Community perspectives and approaches (Mean 3.54, SD 0.70)^a^		
Gender		
Male	Ref.	Ref.
Female	−0.01	−0.17 to 0.14
Others	0.07	−0.63 to 0.77
Age		
24	Ref.	Ref.
25	0.15	−0.03 to 0.32
26	−0.01	−0.22 to 0.20
≥27	−0.07	−0.30 to 0.15
University type		
National or public	Ref.	Ref.
Private	−0.03	−0.18 to 0.13
University location		
Rural	Ref.	Ref.
Urban	−0.07	−0.21 to 0.08
Life perspectives and approaches (Mean 3.52, SD 0.80)^a^		
Gender		
Male	Ref.	Ref.
Female	−0.03	−0.21 to 0.14
Others	−0.04	−0.84 to 0.76
Age		
24	Ref.	Ref.
25	0.19	−0.01 to 0.39
26	0.07	−0.17 to 0.31
≥27	−0.06	−0.31 to 0.20
University type		
National or public	Ref.	Ref.
Private	0.08	−0.10 to 0.26
University location		
Rural	Ref.	Ref.
Urban	0.03	−0.14 to 0.20
Social perspectives and approaches (Mean 3.42, SD 0.74)^a^		
Gender		
Male	Ref.	Ref.
Female	0.04	−0.12 to 0.20
Others	0.07	−0.67 to 0.81
Age		
24	Ref.	Ref.
25	0.07	−0.11 to 0.26
26	0.06	−0.16 to 0.28
≥27	−0.03	−0.27 to 0.21
University type		
National or public	Ref.	Ref.
Private	0.16	−0.00 to 0.32
University location		
Rural	Ref.	Ref.
Urban	0.09	−0.07 to 0.24

Abbreviations: CI, confidence interval; GM, general medicine; Ref, reference category; SD, standard deviation.

^a^ Scores range from 1 to 5, with higher scores indicating that the respondents learned more fully about GM during medical school.

## 4 DISCUSSION

We performed a nationwide cross‐sectional study to investigate how well undergraduate GM education was provided to medical trainees of the 2016 revision MCC generation in Japan. We distributed an anonymous online questionnaire to participants at the beginning of their residency. The questionnaire was developed with reference to 2022 revision MCC. While about half of the participants agreed that they learned enough about GM‐related topics during medical school, of these, behavioral science was suggested to be somewhat underlearned. There were no individual or university factors associated with the degree of GM learning. To our knowledge, this is the first study to elucidate the degree of GM learning from the perspective of medical trainees and will be valuable in the formulation and provision of GM‐related teaching in and outside Japan.

As noted in the Introduction, the inclusion of “generalism” as a new expertise quality and ability in the latest edition of the MCC is based on the results of surveys of experts in health profession education. Thus, it is surprising that half of the medical trainees in this study reported that they learned enough about GM during medical school. In fact, Takamura expressed concern that undergraduate GM education was not sufficiently provided.^30^ Conversely, the Japan Primary Care Association (JPCA), the recognized certifying body for primary care physicians in Japan, conducted a nationwide survey that indicated most medical schools in Japan had a GM department.^31^ Accordingly, even before it was included in the 2022 revision MCC, medical students may have had substantial opportunity to learn about GM. However, the following four possible mechanisms should also be considered. First, medical trainees learned about GM in extracurricular activities. For example, the JPCA conducts a variety of activities, including family medicine seminars for medical trainees, to assist them in choosing a career in GM.^32^ The number of medical students interested in becoming GM physicians has increased recently,^33^ and it is likely that some of these students participated in these extracurricular activities. Second, participants in the GM‐ITE PGY‐0 and our study were likely highly motivated, which could have led to greater scores on the questionnaire responses. Third, the possibility of recall bias remains.^34^ One option for future research might be to administer the survey to medical students prior to their graduation from medical school. However, medical students nearing graduation have a number of significant events (e.g., national license examination for physicians, graduation ceremony) ahead of them, likely prejudicing the response rate to such a survey. Fourth, several items were included in the 2016 revision MCC, although they were not placed in one section as “generalism.” For example, Evidence‐Based Medicine and social sciences related to medical practice were included in “Society and Medicine/Medical Practice” and palliative care in “Basis of Medical Practice.”^35^

Meanwhile, the finding of a perception gap between medical students and faculty members may be consistent with previous research. A Korean study showed a perception gap between medical students and professors regarding the level of professionalism of the medical students.^36^ Metcalf et al. conducted a survey and needs assessment in the obesity medical education that showed that medical students and faculty differed in their perceptions of how well they were prepared in obesity medicine in several areas.^37^ Thus, there tends to be a gap between recipients and providers in their perceptions of medical education, and the perceptions of each stakeholder should be examined.

Our study suggested that behavioral science was likely to be somewhat underlearned among medical trainees. One possible reason is that the content of behavioral science is unfamiliar to medical trainees and thus difficult to understand. A second possible reason is that few faculty members are able to teach behavioral science because they have not been taught it themselves. Behavioral science has now been integrated into undergraduate medical education in a substantial number of countries, including the United States, the United Kingdom, New Zealand, and others.^38^, ^39^, ^40^, ^41^ Conversely, a review article in Japan showed that behavioral science has rarely been treated as an independent curriculum.^42^ Thus, Japanese medical educators should seek to improve behavioral science curricula in light of the 2022 revised MCC.^43^, ^44^ One curriculum suggested by a working group under the auspices of the Japanese Society of Behavioral Medicine consists of 11 units of lectures with 4 units of practical study, which would be insightful for medical educators.^42^

In our study, we could not identify individual or university factors associated with the degree of GM learning. Although such associated factors may not actually exist, it is possible that because of space limitations in the questionnaire we did not include factors that might be associated with the degree of GM learning. For example, as a previous Japanese article indicates, the lack of “real” general practitioners/family physicians in Japan who can teach primary care, including GM, results in medical education being still mainly implemented as an organ‐centered and specialist‐centered curriculum at universities.^30^ Accordingly, future studies should seek information about faculty members who teach GM (e.g., the number of JPCA‐certified family physicians), which would enrich our knowledge of GM education in Japan.

A key strength of our study is its use of data from a nationwide survey. The data covered 74 of the total of 82 medical schools in Japan. In addition, the response rate for the survey was 55.4%, which was above the desirable criterion for online surveys.^45^ Accordingly, our results have relatively high external validity. Another strength of this study is that it collected the perceptions of medical trainees, the primary stakeholders of medical education. The introduction of the “generalism” section of the MCC has tended to focus on the perceptions of those providing medical education in their consideration of the content of medical education. However, the perceptions of other stakeholders are also crucial; while the importance of patient and public involvement has received substantial attention recently, the needs and opinions of the recipients of medical education are also important.^46^, ^47^, ^48^, ^49^ It will be necessary to re‐examine the content of medical education in light of the perception of these often‐neglected stakeholders.

Future studies should survey the generation that received 2022 revision MCC‐compliant undergraduate medical education using the questionnaire we developed and compare the results with those of the present study. Specifically, since the generation that received 2022 revision MCC‐compliant medical education will enter residency in April 2030, a similar survey should be conducted on PGY“‐0” in April 2030. It would also be useful to perform a qualitative study to explore how the experience of receiving 2022 revision MCC‐compliant education is being put to use in the medical field after residency. Such studies will be important in examining the effects of the revised guidelines. Given worldwide concern over undergraduate GM education, the findings of such studies would provide valuable insights to international medical educators.

Potential limitations of this study should be noted. First, as noted above, the possibility of recall bias cannot be excluded. Second, we cannot exclude the presence of social desirability bias. However, we tried our best to minimize this concern (e.g., anonymous survey design). Third, the participants may have had a different medical education as a result of the coronavirus disease 2019 pandemic compared to medical students during the nonpandemic period. Fourth, although we developed our questionnaire with reference to the guidelines for undergraduate medical education, it was not validated. Besides, the broad phrasing of the questions regarding the sufficiency of medical education may limit the specificity of the findings. Further studies should refine the questions to better capture different dimensions of educational adequacy, such as content coverage, practical application, and perceived preparedness, and conduct a validation survey. Fifth, this study had potential selection bias. GM‐ITE PGY‐0 examinees may be highly motivated residents. Since we chose complete case analysis, it is possible that participants with lower learning motivation could have missing data and thus be excluded. We could not include medical trainees from eight universities in Japan, which could also be a potential concern. However, since two‐thirds of each medical school's curriculum should follow the MCC,^22^ it is hard to imagine that the actual curriculum significantly differs from medical school to medical school.

## 5 CONCLUSIONS

In this nationwide cross‐sectional study, we examined how well undergraduate GM education was provided to medical trainees of the 2016 revision MCC generation in Japan. Behavioral science was likely to be somewhat underlearned, whereas approximately half of the participants agreed that they learned enough about other GM‐related topics during medical school. Future studies should survey the generation that received the 2022 revised MCC‐compliant medical education and compare the results with those of our present study; this process would provide crucial information on the effects of the revised guideline and inform medical educators across the world.

## FUNDING INFORMATION

This work was partly supported by the Japan Society for the Promotion of Science, Japan (grant no. JP23K19809).

## CONFLICT OF INTEREST STATEMENT

HT, KS, TS, and YY received an honorarium from the JAMEP as exam preparers of the GM‐ITE. YN received an honorarium from the JAMEP as the GM‐ITE project manager. KS received an honorarium from the JAMEP as a speaker for the JAMEP lecture. YT is the director of JAMEP. HT, YN, KS, TS, YY, and YT were not involved in the data analysis. Otherwise, the authors declare that they have no conflict of interest.

## ETHICS STATEMENT

Ethical approval statement: Ethical approval was granted by the Ethics Review Board of JAMEP (approval number: 23–35).

Patient consent statement: All participants provided informed consent before participating in the study.

Clinical trial registration: None.

## OTHER INFORMATION

YN is an Editorial Board member of Journal of General and Family Medicine. To minimize bias, he was excluded from all editorial decision‐making related to the acceptance of this article for publication.

## Supporting information


**Data S1:** Supporting Information.

## Data Availability

The corresponding author may, upon reasonable request, provide the data sets generated and analyzed in the study.
