# Supplementary material for: Undergraduate general medicine education in Japan: A nationwide cross‐sectional survey of medical trainees' perspectives
Source: J Gen Fam Med. 2024 Nov 10;26(2):148–56. doi: 10.1002/jgf2.752 (PMC11890055; doi:10.1002/jgf2.752)
Supplement: Supplementary file 1 — Data S1: Supporting Information. [file JGF2-26-148-s001.pdf]

Supplementary file: the questionnaire for examining the extent to which the medical trainees felt they had learned about general medicine-related topics (in Japanese)

<u>以下の項目について、医学生時代に十分学習できましたか？</u>

1. 臓器横断的な診療
(1) 強く反対する
(2) 反対する
(3) どちらでもない
(4) 賛成する
(5) 強く賛成する

2. 生物・心理・社会的な問題への包括的な視点
(1) 強く反対する
(2) 反対する
(3) どちらでもない
(4) 賛成する
(5) 強く賛成する

3. 患者中心の医療
(1) 強く反対する
(2) 反対する
(3) どちらでもない
(4) 賛成する
(5) 強く賛成する

4. 根拠に基づいた医療 (EBM)
(1) 強く反対する
(2) 反対する
(3) どちらでもない
(4) 賛成する
(5) 強く賛成する

5. 行動科学
(1) 強く反対する
(2) 反対する
(3) どちらでもない
(4) 賛成する
(5) 強く賛成する

6. 緩和ケア
(1) 強く反対する
(2) 反対する
(3) どちらでもない
(4) 賛成する
(5) 強く賛成する

7. プライマリ・ケアにおける基本概念
(1) 強く反対する
(2) 反対する
(3) どちらでもない
(4) 賛成する
(5) 強く賛成する

8. 地域におけるプライマリ・ケア
(1) 強く反対する
(2) 反対する
(3) どちらでもない
(4) 賛成する
(5) 強く賛成する

9. 医療資源に応じたプライマリ・ケア
(1) 強く反対する
(2) 反対する
(3) どちらでもない
(4) 賛成する
(5) 強く賛成する

10. 在宅におけるプライマリ・ケア
(1) 強く反対する
(2) 反対する
(3) どちらでもない
(4) 賛成する
(5) 強く賛成する

11. 人生のプロセス（ライフサイクルやライフイベント）を意識した診療
(1) 強く反対する

(2) 反対する
(3) どちらでもない
(4) 賛成する
(5) 強く賛成する

12. 医学的・文化的・社会的文脈における健康
(1) 強く反対する
(2) 反対する
(3) どちらでもない
(4) 賛成する
(5) 強く賛成する

13. 社会科学（文化人類学・社会学、主に医療人類学・医療社会学）
(1) 強く反対する
(2) 反対する
(3) どちらでもない
(4) 賛成する
(5) 強く賛成する

医学生時代に、総じて、総合診療に関して十分学習できましたか？
(1) 強く反対する
(2) 反対する
(3) どちらでもない
(4) 賛成する
(5) 強く賛成する
